# Analysis for lipid nutrient differences in the milk of 13 species from a quantitative non-targeted lipidomics perspective

**DOI:** 10.1016/j.fochx.2023.101024

**Published:** 2023-11-23

**Authors:** Yanzhi Wu, Yinggang Sun, Rui Chen, Yanjun Qiao, Qiu Zhang, Qian Li, Xiaowei Wang, Yuan Pan, Siyi Li, Zeying Wang

**Affiliations:** College of Animal Science & Veterinary Medicine, Shenyang Agricultural University, Shenyang 110866, China

**Keywords:** LC-MS/MS, Porcine milk, Mature milk, Chain length, Saturation, Essential fatty acids

## Abstract

Lipids are essential organic components in milk and have been associated with various health benefits for newborns. However, a comprehensive analysis of lipid profiles across multiple species and levels has been lacking. In this study, we employed liquid chromatography-tandem mass spectrometry (LC-MS/MS) to accurately determine the absolute content of lipid molecules. It revealed that ruminants exhibit a higher concentration of short-chain fatty acids compared to non-ruminants. Additionally, we identified ALC (camel), MGH (horse), and DZD (donkey) as species that display similarities to components found in human milk fat. Remarkably, it reveals that porcine milk fat is characterized by long chain lengths, low saturation, and a high proportion of essential fatty acids. PS (22:5_18:2) could potentially serve as a biomarker in porcine milk. These unique characteristics present potential opportunities for the utilization of porcine milk. Overall, our findings provide valuable insights into the lipidomics profiles of milk from different species.

## Highlights

• Non-targeted lipidomics captures more lipids quantitatively.
• Lipid perspective reveals similarities in horse, donkey, camel, and human milk.
• Deciphering distinct lipid profiles in ruminant and non-ruminant milk.
• Comprehensive porcine milk lipid profile.

## Introduction

Human milk is a highly complex biological fluid that has evolved over millions of years, and its composition is optimal for infant growth and development. Breast milk provides infants with essential nutrients, including carbohydrates, lipids, proteins, and minerals, along with many other benefits such as reducing the risk of obesity, diabetes, and pneumonia (Zhao et al., 2022). Unfortunately, some mothers may not be able to produce sufficient breast milk due to genetic, dietary, or other reasons, and infant formula can provide necessary nutrients for the growth of these newborns (Golan & Assaraf, 2020). Currently, cow milk- or soy-based infant formula is the most commonly used industrial substitute for breast milk (Sun et al., 2023). Formula milk producers aim to mimic the components of breast milk, including proteins, fats, and minerals, based on medical and nutritional research findings to achieve optimal benefits. However, due to our current limited understanding of breast milk composition, it's challenging to fully replicate its nutritional components (Martin et al., 2016).

Lipids are a highly variable macronutrient in milk composition, providing between 40 % and 50 % of the total energy for growing infants, despite their low concentration between 3 % and 5 % (w/v) (Liu et al., 2018). Studies suggest that while the overall lipid composition of mammalian milk is similar to human milk (Zou et al., 2013). Differences in total lipid content, fatty acid profiles, and the positional distribution of fatty acids contribute to distinctions between human milk and mammalian milk. Recent research has shown that significant differences in triglyceride composition exist between human milk and formula derived from cow's milk and goat's milk (Sun et al., 2018). These variations are influenced by a key factor: dietary lipids in ruminants undergo almost complete hydrolysis, and unsaturated fatty acids are primarily hydrogenated in the rumen. The rumen's hydrogenation process primarily originates from rumen microorganisms, with its intricate microbial ecosystem playing a pivotal role in the transformation of various lipids. For instance, the L. plantarum YW11 strain demonstrates the bioconversion of linoleic acid (LA) into conjugated linoleic acid (CLA) (Aziz et al., 2022). It should be noted that there are significant variations in milk fat composition among different ruminant animals. For instance, yak milk exhibits relatively higher levels of unsaturated fatty acids compared to cow milk (Li et al., 2023). In contrast, in monogastric animals, dietary fatty acids arrive at the intestine without undergoing modification (Hue-Beauvais et al., 2021). This implies that the degree of lipid molecule saturation is generally higher in ruminant milk. Moreover, ruminant animals typically possess higher levels of short-chain fatty acids (SCFAs). This is due to the complex microbial communities present in their rumen, which utilize plant materials to generate SCFAs and subsequently transfer them into milk (Shen et al., 2019). In early life, SCFAs play pivotal roles in infant energy metabolism, influencing their growth and development (Blaak et al., 2020). Moreover, they contribute to bolstering the integrity of the intestinal barrier, thereby mitigating inflammatory responses in infants grappling with gastrointestinal disorders (Blaak et al., 2020). In comparison, camels, often referred to as pseudo-ruminants due to their three-compartment stomachs rather than four, have higher levels of polyunsaturated fatty acids (PUFAs) (Verduci et al., 2019). PUFA contains various essential fatty acids, such as linoleic acid (LA), α-linolenic acid (ALA), and docosahexaenoic acid (DHA). Approximately 35 % of the lipids in the gray matter of the brain consist of long-chain PUFAs, with DHA and ARA being particularly crucial components of the brain (Einerhand et al., 2023). Long-chain PUFAs are not only critical for the development of the infant's nervous system but also play an indispensable role in shaping the immune system of a developing individual, including resistance to allergic and respiratory diseases (Miles et al., 2021). Donkey milk and horse milk are currently the most common non-ruminant animal milk products on the market. Previous research has indicated that the lipid composition of donkey milk and horse milk is similar, with both being rich sources of ALA and LA (Verduci et al., 2019). Some recent studies have shown that donkey milk and horse milk have PUFA content comparable to human milk, indicating that they can serve as high-quality base ingredients for formula milk (Cimmino et al., 2023, Deng et al., 2022).

Previous studies have provided fairly comprehensive summaries of the lipid profiles across various species (Cimmino et al., 2023). However, a more comprehensive comparison of lipid profiles is needed. While it is possible to compare data from different articles, the low abundance and highly dynamic nature of lipids, along with the random errors introduced by different analytical environments and methods, cannot be ignored across different studies (Li et al., 2021). Therefore, a comprehensive analysis encompassing a wide range of species and utilizing standardized methods would be meaningful.

Lipidomics is a specialized field within the broader realm of metabolomics that focuses on the identification and quantification of lipid molecular species. In recent years, lipidomics has gained recognition as a distinct field due to its unique analytical challenges and the importance of lipids in various biological processes (R. Wang et al., 2020). Mass spectrometry coupled with liquid chromatography (LC-MS) is the most commonly used technique for lipidomics analysis in milk (Liu et al., 2018). While MS is a destructive technique that precludes future analysis of the sample, its high sensitivity and specificity enable the identification and structural characterization of known and unknown lipids. LC-MS can provide detailed information on the fatty acid composition of each lipid, the regiospecific location of fatty acids, and quantitative data on individual lipid species (Liu et al., 2018). Previous research comparing lipid composition between animal and human milk has focused primarily on a limited number of common mammals including: cow, goat, sheep, donkey, mare, camel (L. Wang et al., 2020). In this study, we employed LC-MS/MS non-target metabolism techniques to analyze differential lipids in the milk of 13 mammalian species. We then annotated these lipids with LipidSearch version 4.2 to identify additional lipids, providing novel insights into the diversity and content of milk lipids. Our findings may have implications for the development of the dairy industry.

## Materials and methods

### Test sample collection

We collected thirteen mammalian milk samples for this experiment, six replicates of each specie, as follows: Chinese human milk samples (CHP) were donated by mothers at the Shenyang Maternal and Child Care Center. Holstein cow milk samples (HST) were sourced from Huishan Dairy Group. Buffalo milk samples (GXB) were sourced from the Guangxi Buffalo Research Institute and Nanjing Agricultural University. Yak milk samples (QHY) were sourced from Qinghai Province and the Lanzhou Institute of Husbandry and Pharmaceutical Science of Cass. DeZhou Donkey milk samples (DZD) were sourced from the East Ajiao Donkey Farm in Fumeng County. Mongolian Horse milk samples (MGH) were sourced from the Inner Mongolia XilinGol League Science and Technology Association. Alxa camel milk samples (ALC) were sourced from Alxa Alpaca Farm in Inner Mongolia. Saanen milk goat milk samples (SNG), Toggenburg milk goat milk samples (TGB), and Nubian milk goat milk samples (NBY) were sourced from Liaoyang Dairy Goat Farm. Liaoning Cashmere goat milk samples (LCG) were sourced from Liaoyang National Core Sheep Breeding Farm. Small Tail Han sheep milk samples (OAS) were sourced from Inner Mongolia Horinger County Sheep Farm. Pig milk samples (YXP) were sourced from Yangxiang Pig Group. All samples were collected in the fall from healthy individuals. The information of experimental animals was shown in Table S1. In all species, milk samples were collected within the second lactating week after the birth of the second offspring. At the end of each feeding or milking, the milk sample is removed and placed in the same 10 m L plastic container and immediately placed in a refrigerator at −20 °C for a short period of time, not exceeding two weeks. The samples are then transported in undefrosted dry ice and placed in a freezer at −80 °C for long-term storage, but for no more than four months. The samples were collected and tested at the same time.

**Ethical statements**: Ethical approval for the involvement of human subjects in this study was granted by Shenyang Agricultural University Research Ethics Committee, Reference number 202106015.

The collection of human milk samples was conducted with the informed consent of the individuals and their family members. The sample collection was carried out with the assistance of a medical professional. The collection of animal samples was performed with the consent of the farm owner and under the guidance of professional technicians to minimize animal stress responses.

### Chemicals and reagents

MS-grade methanol, MS-grade acetonitrile, HPLC-grade 2-propanol were purchased from Thermo Fisher, USA. HPLC-grade formic acid and HPLC-grade ammonium formate were purchased from Sigma, USA.

The absolute quantification of lipids is based on the internal standard method: the response abundance ratio (primary peak area ratio) of the substance that is measured and the concentration of the internal standard (1 mg/mL) are used to calculate the absolute content of the substance that is measured. The internal standards (SPLASH® LIPIDOMIX® Mass Spec Standard, Avanti, 330707-1EA.): ceramide (Cer), lysophosphatidylcholine (LPC), phosphatidylcholine (PC), lysophosphatidylethanolamine (LPE), phosphatidylethanolamine (PE), phosphatidylinositol (PI), phosphatidylserine (PS), PA, phosphatidylglycerol (PG), sphingomyelin (SM), Chol Ester (CE), diphosphatidylglycerol (DG), triglyceride (TG).

### Sample preparation and lipid extraction

Lipids were extracted according to methyl-*tert*-butyl ether (MTBE) method. Briefly, samples were first spiked with appropriate amount of internal lipid standards and then homogenized with 200 µ L water and 240 µ L methanol. After that, 800 µ L of MTBE was added and the mixture was ultrasound 20 min at 4 ℃ followed by sitting still for 30 min at room temperature. The solution was centrifuged at 14000g for 15 min at 10 ℃ and the upper organic solvent layer was obtained and dried under nitrogen.

### Mass spectrometry

Analyses were performed using an UHPLC Nexera LC-30A ultra performance liquid chromatography system (SHIMADZU, Japan) coupled to Q-Exactive Plus (Thermo Scientific) in Shanghai Applied Protein Technology Co., Ltd.

#### High performance liquid chromatography (HPLC)

For LC-MS/MS method for lipid analysis, reverse phase chromatography was selected for LC separation using CSH C18 column (1.7 μm, 2.1 mm × 100 mm, Waters). The lipid extracts were re-dissolved in 200 μL of 90 % isopropanol/acetonitrile, centrifuged at 14,000 × g for 15 min, and finally 3 μL of sample was injected. Solvent A was acetonitrile–water (6:4, vol/vol) with 0.1 % formic acid and 0.1 mM ammonium formate, and solvent B was acetonitrile–isopropanol (1:9, vol/vol) with 0.1 % formic acid and 0.1 mM ammonium formate. The initial mobile phase was 30 % solvent B at a flow rate of 300 μL/min. It was held for 2 min, and then linearly increased to 100 % solvent B in 23 min, followed by equilibrating in 5 % solvent B for 10 min. Mass spectra was acquired by Q-Exactive Plus in positive and negative modes, respectively. ESI (Electron Spray Ionization) parameters were optimized and preset for all measurements as follows: source temperature, 300 °C; capillary temperature, 350 °C; the ion spray voltage was set at 3000 V, S-Lens RF Level was set at 50 % and the scan range of the instruments was set at 200–1800 *m*/*z*. Mass spectra was acquired by Q-Exactive Plus in positive and negative mode, respectively. ESI parameters were optimized and preset for all measurements as follows: Source temperature, 300 °C; Capillary Temp, 350 °C, the ion spray voltage was set at 3000 V, S-Lens RF Level was set at 50 % and the scan range of the instruments was set at *m*/*z* 200–1800 (Liu et al., 2022).

#### Tandem mass spectrometry (MS/MS) analysis

Electrospray ionization (ESI) was used to detect positive and negative ions. The samples were separated by UHPLC and analyzed by a Q exactive mass spectrometer (Thermo Fisher Scientific, USA). The ESI source conditions are as follows: heater temperature 300 °C, sheath gas flow rate 45 arb, aux gas flow rate 15 arb, sweep gas flow rate 1 arb, spray voltage 3.0 KV, capillary temperature 350 °C, S-Lens RF level 50 %, MS1 scan ranges: 200–1800. The mass charge ratio of lipid molecules and lipid fragments was collected according to the following methods: 10 fragments (MS2scan, HCD) were collected after each full scan. The resolution of MS1 is 70,000 at *m*/*z* 200 and that of MS2 is 17,500 at *m*/*z* 200.

### Data processing and statistical analysis

“Lipid Search” is a search engine for the identification of lipid species based on MS/MS math. Lipid Search contains more than 30 lipid classes and more than 1,500,000 fragment ions in the database. Both mass tolerance for precursor and fragment were set to 5 ppm. Lipid species were identified using the LipidSearch software version 4.2 (Thermo Scientific™) to process the raw data and for peak alignment, retention time correction and extraction peak area. Adducts of + H, +NH_4_ were selected for positive mode searches, and -H, +CH_3_COO were selected for negative mode searches since ammonium acetate was used in the mobile phases. For the data extracted from LipidSearch, remove the ion peak with a value of > 50 % missing from the group. After normalization and integration using the Perato scaling method, the processed data were imported into SIMPCA-P 16.1 (Umetrics, Umea, Sweden) for a multivariate statistical analysis, including a principal component analysis (PCA), partial least squares discriminant analysis (PLS-DA), and orthogonal partial least squares discriminant analysis (OPLS-DA). Lipids with significant differences were identified based on a combination of statistically significant thresholds of variable influence on projection (VIP > 1) values obtained from the OPLS-DA model (mutidi-mensional statistical analysis) and two-tailed student’s *t*-test (P-value < 0.05) on the raw data (unidimensional statistical analysis) by PASS 16 (https://www.ncss.com/software/pass/) before experiments.

## Result

### Reliability of the analytical method

Several statistical methods were employed in this study to evaluate the quality and reliability of the lipidomics data. These methods included the comparison of Base Peak Spectra (BPC) of quality control (QC) samples, principal component analysis (PCA) of the overall samples, Hotelling's T2 test of the overall samples, and a multivariate control chart of the QC samples. To assess the stability, repeatability, and overall data quality, the relative standard deviation (RSD) of the QC samples served as a comprehensive indicator. Following six replicate experiments, the data were deemed reliable and suitable for further analysis (Refer to the 'Quality Control' document provided in the supplementary materials).

### Statistics of identification numbers

**Fig. 1:**
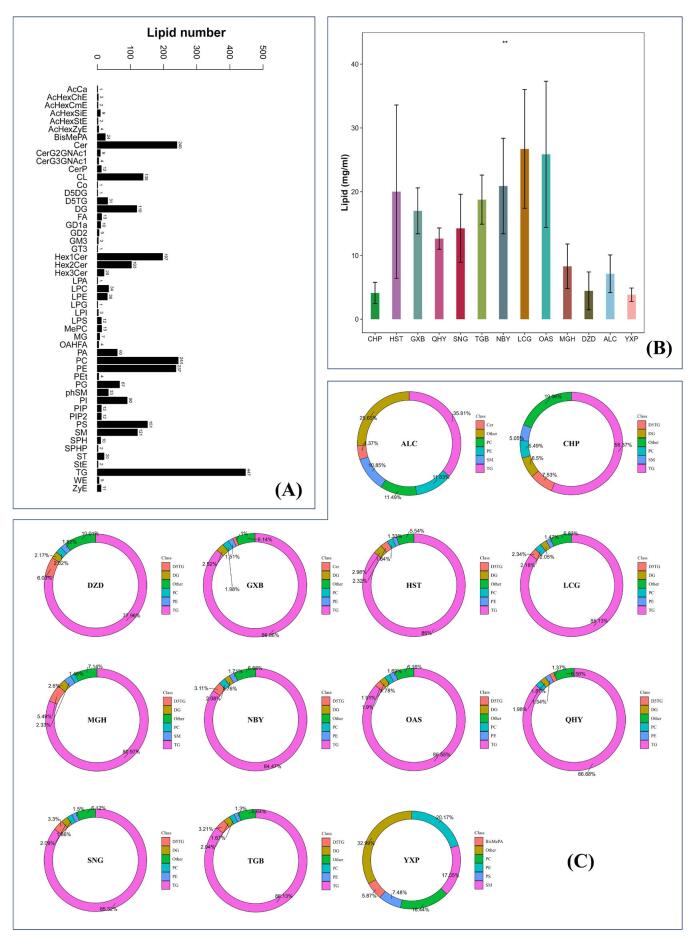
(A) Bar chart of lipid subclass count; (B) Bar chart of lipid content in different species; (C) donut chart of the composition of lipid subclasses in different species.

As shown in Fig. 1(A), the analysis revealed a total of 2585 lipids belonging to 51 distinct classes, detected in both positive (POS) and negative (NEG) ion modes. Among these lipids, a subset of 13 lipid classes displayed more than 50 identifications each. These lipid classes included Ceramides (Cer), Cardiolipin (CL), diglyceride (DG), Monoglycosylceramide (Hex1Cer), Diglycosylceramide (Hex2Cer), phosphatidic acid (PA), phosphatidylcholine (PC), phosphatidylethanolamine (PE), phosphatidylglycerol (PG), phosphatidylinositol (PI), phosphatidylserine (PS), sphingomyelin (SM), and triglyceride (TG).

### Analysis of total lipid content and composition of different species

The total lipid content of human (CHP), pig (YXP), donkey (DZD), horse (MGH), and camel (ALC) was found to be lower than that of buffalo (GXB), cow (HST), Liaoning cashmere goat (LCG), Nubian goat (NBY), Saanen goat (SNG), Toggenburg goat (TGB), sheep (OAS), and Yak (QHY), as depicted in Fig. 1(B). Excluding YXP, the total lipid content in all other mammals was found to be higher than that in CHP Fig. 1(C) presents the lipid composition of each species under investigation. Notably, the distribution of lipid subclasses exhibited significant variations among the milk samples. In CHP, the top five lipid subclasses in terms of content are TG, Deuterated triglyceride (D5TG), DG, PC, and SM. Compared to CHP, all samples, except for ALC and YXP, exhibited a higher proportion of TG. ALC and YXP, on the other hand, had higher proportions of PC, PE, and SM compared to CHP. With the exception of YXP, most species demonstrated a higher percentage of triglycerides as the dominant subclass. Specifically, excluding ALC and YXP, exhibited TG content exceeding 50 %. Apart from TG, ALC showed significant proportions of PE, PC, and SM, accounting for more than 30 % of the lipid composition. YXP stood out as the most distinctive species, with PC, SM, and PE being the major lipids, collectively contributing to over 53 % of the total lipid content.

### Multidimensional statistical analysis

**Fig. 2:**
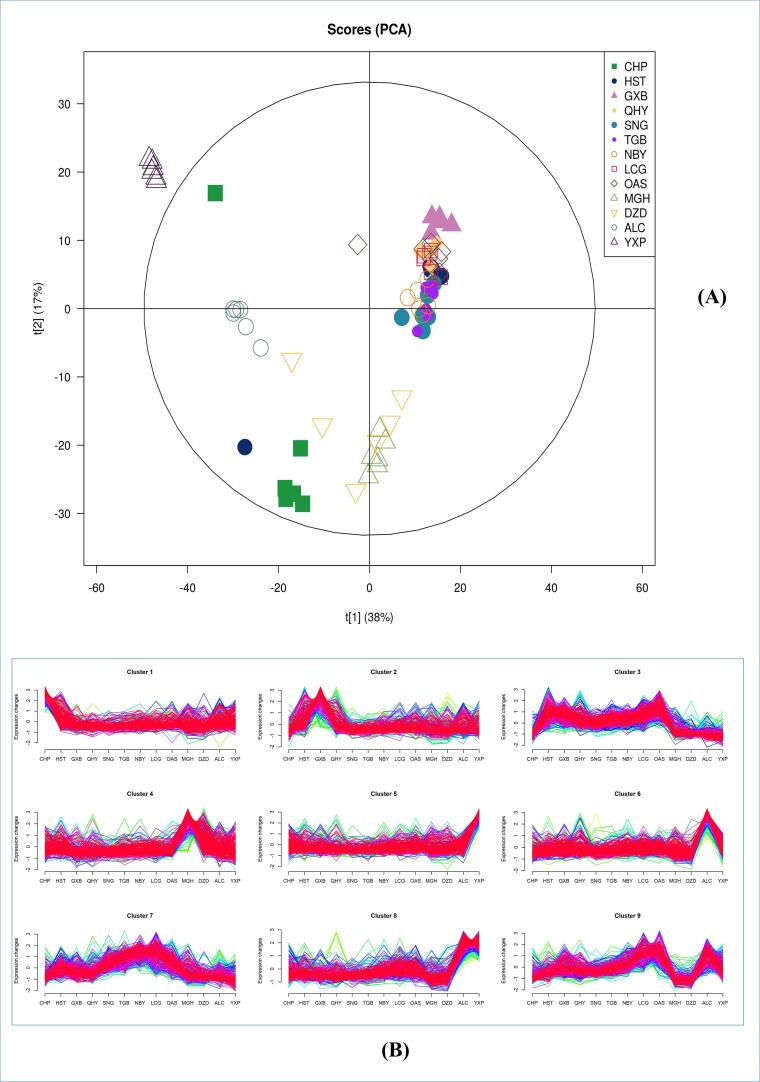
(A) Principal component analysis; (B) Trend clustering chart.

Based on the outcomes of principal component analysis (PCA), the first two principal components (PC1 and PC2) were selected to generate a PCA scatterplot. The cumulative contribution rate of PC1 and PC2 accounted for 55 % of the total variance. Fig. 2(A) depicts a clear separation of CHP, ALC, YXP, and MGH from the other species. The objects HST, GXB, QHY, SNG, NBY, TGB, LCG, and OAS displayed significant clustering tendencies. Consequently, the 13 sample groups were categorized into five distinct classes. Specifically, CHP, YXP, and ALC formed three separate classes, while MGH and DZD constituted one class. Additionally, HST, GXB, QHY, SNG, NBY, TGB, LCG, and OAS belonged to a single class. It is noteworthy that the samples with the lipid composition closest to that of CHP are DZD and MGH.

### Trend cluster analysis

Trend cluster analysis was conducted to explore the abundance patterns and trends of the identified lipid molecules within each experimental group. The Mfuzz software, utilizing the fuzzy c-means (FCM) algorithm, was employed to partition the data into distinct abundance modules based on the overall lipid profile. The resulting clustering outcomes are visualized in Fig. 2(B).

The analysis revealed notable patterns among the different clusters. Cluster1 exhibited a high abundance of CHP, while Cluster2 was characterized by enrichment in HST, GXB, and QHY. Cattle and sheep were predominantly grouped in Cluster3, with relatively lower abundance of MGH, DZD, and ALC. Cluster4 displayed a high abundance of MGH and DZD. YXP was assigned to Cluster5, whereas ALC showed high abundance in both Cluster5 and Cluster6. Cluster7 comprised OAS, LCG, NBY, SNG, TGB. Cluster8 exhibited a high abundance of ALC and YXP, while MGH and DZD had lower abundance within this cluster. Cluster9 displayed a high abundance of LGC, OAS, and ALC, with lower abundance of MGH and DZD.

Further analysis of the lipid distribution pattern within each species revealed distinct characteristics of different clusters. In Cluster1, lipids with long carbon chain lengths and high polyunsaturated fatty acid content, particularly 16-20C fatty acids, were prominent. Cluster2 and Cluster3 exhibited high lipid saturation, with Cluster3 specifically enriched in TG molecules containing short- and medium-chain fatty acids (less than 10 carbon atoms). Cluster4 also displayed enrichment in TG molecules, but with lower saturation compared to Cluster3. Clusters 5, 6, 8, and 9 were primarily enriched in Cer and CL, with a lower abundance of TG. Cluster5 demonstrated longer carbon chain lengths and higher unsaturation, while Cluster6 exhibited fatty acid enrichment with 14–18 carbon atoms. Cluster7 mainly comprised lipids composed of some short-chain fatty acids.

To further elucidate the PCA results, trend clustering analysis was employed to investigate the lipid distribution patterns of CHP, DZD, and MGH. Within clusters 3, 5, 6, 8, and 9, CHP, DZD, and MGH exhibited similar trends. Cluster 3 displayed characteristics with high saturation, while clusters 5, 6, 8, and 9 featured low TG and high Cer and CL content. It is noteworthy that CHP had a high presence in cluster 1, indicating its segregation from other samples in lipids characterized by long chains and low saturation. Cluster 4, on the other hand, represented a common feature of DZD and MGH, featuring lipids with short chains and low saturation, which may signify a unique lipid distribution pattern for DZD and MGH.

### Differential lipid analysis

Based on the univariate analysis, a differential analysis was conducted on all detected lipid molecules, and the results were visualized in a multi-group difference volcano map. In the multi-group difference volcano map, all samples were compared to the CHP sample. Differential lipid molecules with a Fold Change (FC) > 2 or FC < 0.5 and a p-value less than 0.05 are highlighted in red, while those with p-values > 0.05 are marked in blue.

**Fig. 3:**
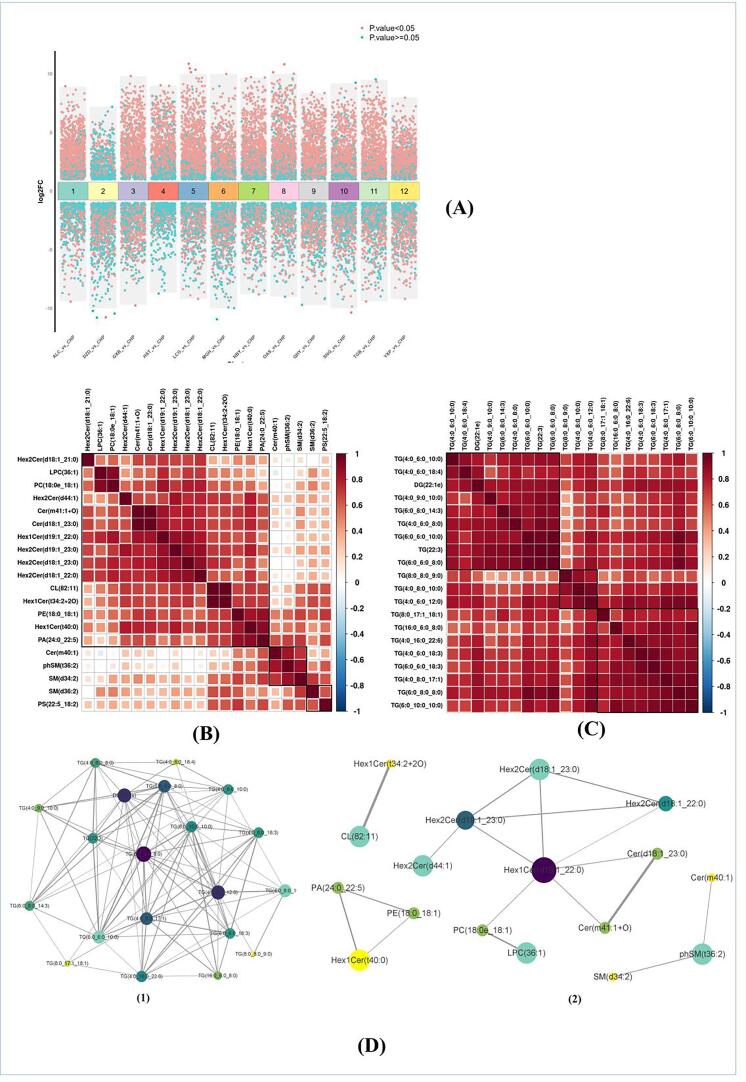
(A) Multi-group difference analysis volcano plot; (B) Correlation heatmap of top 20 differential lipid molecules in negative ion mode; (C) Correlation heatmap of top 20 differential lipid molecules in positive ion mode; (D) Network diagram of the top 20 differential lipid molecules in both positive and negative ion modes.

As shown in Fig. 3(A), DZD (237up/576down), and MGH (517up/689down) exhibited a higher number of down-regulated lipids compared to up-regulated ones. Conversely, ALC (1048up/236down), GXB (975up/412down), HST (797up/168down), QHY (871up/436down), LCG (992up/306down), NBY (899up/342down), SNG (771up/442down), TGB (960up/381down), OAS (934up/303down), and YXP (889up/418down) showed a higher number of up-regulated lipids compared to down-regulated ones. This result aligns with the PCA findings, with MGH and DZD emerging as the three closest groups to CHP. Both MGH and DZD are non-ruminant animals, but they possess certain differences in their digestive systems compared to humans, which may account for the differential lipid profiles. Except for YXP, most groups with more upregulated lipids than downregulated lipids are ruminants. This implies that ruminant milk may have unique lipid secretion characteristics compared to CHP. YXP represents the most distinct category within the samples and was also classified as a separate group in the PCA results. Combining PCA analysis and trend clustering analysis, the origin of the upregulated lipids in YXP is likely associated with a high content of polyunsaturated long-chain fatty acids. This hypothesis will need validation in subsequent analyses.

### The top 20 lipids with the highest differences in positive and negative ion mode

In this study, a total of 805 differential lipids (P value < 0.05) were identified in the positive ion mode, while 1666 differential lipids were identified in the negative ion mode. Combined, a total of 2471 differential lipids were obtained. Fig. S1 and Table 1 present the top 20 differential lipids in both the positive and negative ion modes.

**Table 1:** The top 20 differential lipid molecules in both positive and negative ion modes.

	CHP	HST	GXB	QHY	SNG	TGB	NBY	LCG	OAS	MGH	DZD	ALC	YXP
SM(d36:2)	0.84 ± 0.45	0.69 ± 0.18	0.06 ± 0.02	0.12 ± 0.07	1.26 ± 0.35	1.28 ± 0.46	2.56 ± 1.82	1.45 ± 0.94	1.37 ± 0.76	0.01 ± 0.01	0.01 ± 0	0.92 ± 0.38	1.33 ± 0.21
Hex2Cer(d18:1_23:0)	0.06 ± 0.06	1.01 ± 0.66	4.67 ± 1.33	2.13 ± 1.01	2.7 ± 1.12	3.84 ± 2.03	5.86 ± 2.68	4.62 ± 1.81	2.29 ± 1.5	0.01 ± 0	0.02 ± 0.02	2.65 ± 0.56	0.03 ± 0.03
phSM(t36:2)	0.23 ± 0.17	0.34 ± 0.16	0.08 ± 0.03	0.25 ± 0.26	0.23 ± 0.08	0.26 ± 0.12	0.37 ± 0.2	0.46 ± 0.16	0.26 ± 0.08	0.01 ± 0.01	0.01 ± 0	1.48 ± 0.56	2.32 ± 0.72
Hex2Cer(d18:1_22:0)	0.23 ± 0.13	1.31 ± 0.71	2.34 ± 0.83	1.5 ± 0.58	2.67 ± 0.79	2.88 ± 1.38	2.99 ± 0.78	1.66 ± 0.31	0.92 ± 0.51	0.05 ± 0.01	0.09 ± 0.06	3.36 ± 1.01	0.16 ± 0.11
SM(d34:2)	0.37 ± 0.17	0.84 ± 0.42	0.23 ± 0.08	0.42 ± 0.35	0.52 ± 0.13	0.75 ± 0.27	1.31 ± 1.09	1.95 ± 1.55	0.82 ± 0.53	0.02 ± 0.01	0.01 ± 0	2.54 ± 1.51	2.67 ± 1.16
LPC(36:1)	0.27 ± 0.12	1.7 ± 1.3	1.13 ± 0.43	0.47 ± 0.15	2.16 ± 0.91	1.59 ± 0.69	1.71 ± 1.1	0.97 ± 0.17	0.92 ± 0.2	0.03 ± 0.01	0.02 ± 0.02	0.59 ± 0.18	0.25 ± 0.09
CL(82:11)	0.07 ± 0.03	0.31 ± 0.25	0.57 ± 0.12	1.03 ± 0.23	0.86 ± 0.18	1.53 ± 0.45	2.63 ± 1.92	2.55 ± 2.66	2.09 ± 1.32	0.02 ± 0.02	0.02 ± 0.01	1.47 ± 0.59	0.7 ± 0.17
Hex1Cer(t40:0)	0.93 ± 0.3	2.22 ± 1.05	3.84 ± 0.97	3.82 ± 1.37	3.87 ± 1.46	3.64 ± 1.41	4.58 ± 1.66	5.15 ± 2.21	6.84 ± 3.26	0.21 ± 0.1	0.35 ± 0.21	17.68 ± 5.06	3.48 ± 0.92
Hex2Cer(d44:1)	0.01 ± 0.01	0.01 ± 0.01	0.09 ± 0.04	0.03 ± 0.02	0.1 ± 0.05	0.09 ± 0.04	0.14 ± 0.07	0.14 ± 0.06	0.09 ± 0.04	0 ± 0	0 ± 0	0.19 ± 0.06	0.02 ± 0.02
Hex2Cer(d18:1_21:0)	0.02 ± 0.02	0.5 ± 0.31	0.82 ± 0.43	0.51 ± 0.19	0.29 ± 0.09	0.29 ± 0.1	0.4 ± 0.14	0.36 ± 0.13	0.11 ± 0.02	0.01 ± 0	0.01 ± 0.01	0.37 ± 0.11	0.19 ± 0.06
PE(18:0_18:1)	32.52 ± 10.78	41.3 ± 20.9	47.29 ± 16.47	46.59 ± 16.88	36.74 ± 14.02	40.22 ± 13.89	59.69 ± 26.78	82.71 ± 30.7	110.8 ± 45.68	2.57 ± 1.77	3.28 ± 2.6	123.94 ± 34.87	86.02 ± 31.03
Cer(m40:1)	0.12 ± 0.07	0.07 ± 0.02	0.03 ± 0.03	0.14 ± 0.11	0.03 ± 0.01	0.05 ± 0.03	0.07 ± 0.06	0.06 ± 0.04	0.05 ± 0.01	0 ± 0	0 ± 0	0.45 ± 0.28	0.37 ± 0.15
Hex1Cer(d19:1_22:0)	0.18 ± 0.11	3.21 ± 2.39	6.93 ± 1.97	2.32 ± 1.06	4.65 ± 2.7	4.05 ± 2.01	4.94 ± 2.65	5.17 ± 3.85	3.09 ± 1.44	0.04 ± 0.01	0.04 ± 0.03	4.02 ± 0.86	0.39 ± 0.14
Cer(m41:1 + O)	0.41 ± 0.19	5.37 ± 4.45	13.04 ± 9.22	6.15 ± 2.27	3.04 ± 1.57	4.23 ± 1.88	6.3 ± 4.39	10.23 ± 6.43	9.53 ± 3.58	0.08 ± 0.04	0.11 ± 0.07	15.22 ± 8.78	0.89 ± 0.36
PA(24:0_22:5)	0.97 ± 0.32	1.35 ± 0.31	1.73 ± 0.59	3.12 ± 1.1	1.78 ± 0.46	2.06 ± 0.83	2.31 ± 0.6	3.02 ± 0.99	5.07 ± 2.41	0.08 ± 0.06	0.33 ± 0.32	9.21 ± 4.95	4.14 ± 1.16
Hex2Cer(d19:1_23:0)	0.33 ± 0.34	0.66 ± 0.27	2.92 ± 0.9	1.07 ± 0.44	1.98 ± 0.83	2.14 ± 1.11	2.68 ± 0.79	2.39 ± 1.31	1.34 ± 0.24	0.02 ± 0.01	0.09 ± 0.08	2.56 ± 0.43	0.79 ± 0.61
PS(22:5_18:2)	0.14 ± 0.05	0.22 ± 0.05	0.24 ± 0.05	0.39 ± 0.09	0.33 ± 0.13	0.4 ± 0.13	0.41 ± 0.14	0.38 ± 0.04	0.46 ± 0.18	0.02 ± 0.02	0.03 ± 0.02	0.24 ± 0.06	0.79 ± 0.11
Hex1Cer(t34:2 + 2O)	0.14 ± 0.08	0.34 ± 0.25	0.55 ± 0.12	1.04 ± 0.22	0.89 ± 0.2	1.56 ± 0.42	2.69 ± 1.92	2.62 ± 2.65	2.38 ± 1.34	0.02 ± 0.02	0.02 ± 0.02	1.55 ± 0.66	0.71 ± 0.17
PC(18:0e_18:1)	0.47 ± 0.16	2.56 ± 1.99	2.21 ± 0.65	0.84 ± 0.25	3.37 ± 1.89	3.2 ± 1.72	4 ± 3.21	2 ± 0.7	1.1 ± 0.31	0.07 ± 0.02	0.04 ± 0.04	1.94 ± 0.52	0.7 ± 0.27
Cer(d18:1_23:0)	0.47 ± 0.22	6.47 ± 5.21	14.23 ± 8.69	9.45 ± 2.73	3.98 ± 1.85	4.7 ± 1.72	7.28 ± 5.21	10.75 ± 6.85	10.98 ± 4.45	0.11 ± 0.06	0.14 ± 0.08	16.4 ± 9.48	0.87 ± 0.35
TG(6:0_6:0_10:0)	0.01 ± 0.02	0.46 ± 0.34	1.55 ± 0.49	1.09 ± 0.73	2.05 ± 1.73	2.45 ± 1.31	4.44 ± 2.29	6.18 ± 2.56	4.77 ± 3.57	0.1 ± 0.08	0.03 ± 0.04	0 ± 0	0.01 ± 0
TG(4:0_8:0_10:0)	0.03 ± 0.05	1.55 ± 1.38	24.95 ± 16.25	2.49 ± 1.72	1.55 ± 1.4	3.55 ± 2.76	3.51 ± 2.1	6.32 ± 3.37	12.08 ± 15.25	0.03 ± 0.02	0.01 ± 0.01	0.01 ± 0	0 ± 0
TG(6:0_8:0_14:3)	0 ± 0	0.2 ± 0.13	0.19 ± 0.1	0.36 ± 0.19	0.67 ± 0.79	0.56 ± 0.17	0.86 ± 0.61	2.06 ± 1.07	1.99 ± 1.79	0.01 ± 0.01	0 ± 0.01	0 ± 0	0 ± 0
TG(6:0_8:0_8:0)	0.05 ± 0.11	4.69 ± 2.97	19.96 ± 9.89	7.91 ± 5.23	23.04 ± 21.09	24.84 ± 10.07	44.16 ± 24.5	76.39 ± 37.5	60.51 ± 55.26	0.42 ± 0.28	0.37 ± 0.48	0.01 ± 0	0.01 ± 0.01
TG(22:3)	0 ± 0	0.17 ± 0.14	0.54 ± 0.25	0.42 ± 0.3	0.97 ± 0.93	1.08 ± 0.43	2.01 ± 1.21	3.36 ± 1.95	3.06 ± 2.78	0.01 ± 0.01	0.01 ± 0.01	0 ± 0	0 ± 0
TG(4:0_9:0_10:0)	0.01 ± 0	0.05 ± 0.03	0.17 ± 0.06	0.06 ± 0.03	0.22 ± 0.22	0.47 ± 0.28	0.67 ± 0.39	0.69 ± 0.31	0.37 ± 0.33	0.01 ± 0.01	0 ± 0	0 ± 0	0.01 ± 0
TG(4:0_6:0_18:3)	0.03 ± 0.03	3.6 ± 2.61	3.38 ± 1.75	6.46 ± 4.09	2.27 ± 2.07	3.77 ± 1.63	6.59 ± 3.12	4.24 ± 2.47	5.44 ± 4.16	0.48 ± 0.38	0.03 ± 0.03	0 ± 0	0 ± 0
TG(4:0_16:0_22:6)	0.44 ± 0.36	6.29 ± 4.99	8.71 ± 3.1	16.55 ± 4.41	5.75 ± 2.02	8.77 ± 3.63	9.09 ± 4.49	8.93 ± 3.64	22.79 ± 12.88	0.11 ± 0.07	0.08 ± 0.05	0.48 ± 0.38	0.04 ± 0.02
TG(4:0_6:0_12:0)	0.07 ± 0.15	4.74 ± 3.44	38.32 ± 19.48	7.07 ± 4.69	6.36 ± 5.19	9.54 ± 4.79	13.99 ± 6.56	22.31 ± 8.62	39.32 ± 34.58	0.13 ± 0.11	0.08 ± 0.13	0.01 ± 0	0.01 ± 0
TG(6:0_6:0_8:0)	0.02 ± 0.03	0.68 ± 0.49	1.96 ± 0.96	1.58 ± 1.15	3.72 ± 3.76	4.11 ± 1.72	8.4 ± 5.98	13.86 ± 6.54	13.71 ± 15.18	0.06 ± 0.05	0.04 ± 0.06	0 ± 0	0.01 ± 0
TG(6:0_6:0_18:3)	0.06 ± 0.06	7.67 ± 5.26	6.1 ± 2.51	12.06 ± 6.55	6.97 ± 5.33	12.24 ± 5.14	21.64 ± 10.37	17.5 ± 9.45	13.94 ± 10.48	4.12 ± 3.25	0.33 ± 0.31	0.02 ± 0.01	0.01 ± 0
TG(8:0_17:1_18:1)	0.01 ± 0.02	1.45 ± 1.13	1.21 ± 0.26	1.38 ± 0.57	1.12 ± 0.62	1.36 ± 0.72	1.84 ± 1.44	3.63 ± 1.59	2.65 ± 1.12	0.08 ± 0.05	0.08 ± 0.09	1.11 ± 0.46	0 ± 0
TG(4:0_6:0_10:0)	0 ± 0	0.11 ± 0.1	0.76 ± 0.52	0.12 ± 0.11	0.24 ± 0.17	0.17 ± 0.09	0.62 ± 0.43	1.07 ± 0.42	1.24 ± 1.07	0.01 ± 0.01	0.01 ± 0	0 ± 0	0.01 ± 0.01
DG(22:1e)	0 ± 0	0.47 ± 0.34	0.38 ± 0.15	0.27 ± 0.16	0.42 ± 0.37	0.58 ± 0.27	0.99 ± 0.61	0.67 ± 0.39	1.18 ± 1.05	0.01 ± 0.01	0.01 ± 0.01	0 ± 0	0 ± 0
TG(6:0_10:0_10:0)	0.21 ± 0.48	16.9 ± 10.8	34.09 ± 10.9	15.43 ± 7.07	29.71 ± 20.28	39.1 ± 15.47	41.19 ± 13.22	49.63 ± 24.04	55.65 ± 31.81	2.03 ± 1.22	1.43 ± 2.11	0.01 ± 0.01	0.01 ± 0.01
TG(8:0_8:0_9:0)	0.01 ± 0.01	0.17 ± 0.15	2.32 ± 0.84	0.78 ± 0.43	0.17 ± 0.14	0.49 ± 0.45	0.51 ± 0.38	0.36 ± 0.16	1.82 ± 1.01	0.01 ± 0.01	0 ± 0	0 ± 0	0 ± 0
TG(4:0_8:0_17:1)	0.05 ± 0.12	18.08 ± 13.41	14.85 ± 5.02	9.05 ± 4.48	8.56 ± 5.67	13.32 ± 6.39	22.34 ± 14.02	32.25 ± 13.73	27.93 ± 23.55	0.95 ± 0.43	0.32 ± 0.54	0.01 ± 0	0 ± 0
TG(4:0_6:0_8:0)	0 ± 0	0.12 ± 0.08	0.14 ± 0.08	0.15 ± 0.11	0.32 ± 0.31	0.37 ± 0.17	0.78 ± 0.5	1.23 ± 0.54	1.84 ± 2.49	0.01 ± 0.01	0.01 ± 0	0 ± 0	0 ± 0
TG(4:0_6:0_18:4)	0 ± 0	0.2 ± 0.16	0.39 ± 0.23	0.24 ± 0.18	0.12 ± 0.12	0.13 ± 0.05	0.27 ± 0.24	0.39 ± 0.19	0.86 ± 0.87	0.01 ± 0.01	0 ± 0.01	0 ± 0	0 ± 0
TG(16:0_6:0_8:0)	0.63 ± 1.34	31.18 ± 23.02	29.28 ± 7.89	21.38 ± 7.58	30.11 ± 12.64	33.67 ± 11.48	33.38 ± 10.37	38.84 ± 10.32	37.19 ± 13.7	8.92 ± 2.87	6.87 ± 5.85	0.17 ± 0.06	0.04 ± 0.03

In the positive ion mode, the top 20 lipids with the largest differences primarily belonged to the TG and DG categories. TG and DG are among the top three lipid components in CHP, suggesting their potential as key lipid molecules for distinguishing between milk from different species. These lipid molecules exhibited a notable abundance of medium- and short-chain fatty acids. While these lipid molecules are present at very low levels in CHP samples, they are found in relatively higher amounts in HST, GXB, QHY, SNG, TGB, NBY, LCG, and OAS. This indicates that these lipid molecules have the potential to serve as biomarkers for identifying traditional ruminant milk.

In the negative ion mode, the top 20 lipids with the highest differences included Cer, CL, Hex1Cer, Hex2Cer, LPC, PE, PA, phSM, and SM. While these polar lipid molecules are not the main constituents of milk lipids, their low abundance can often be overshadowed by high-abundance molecules, making it necessary to separately emphasize the top 20 differential lipids in negative ion mode. Among these lipids, Cer (m40:1) showed higher abundance in ALC and YXP compared to other species. DZD and MGH displayed lower abundance of these lipids. CHP has relatively higher content in Cer (m40:1), PE (18:0_18:1), and PS (22:5_18:2). In contrast, ALC and YXP exhibit significantly higher levels of these lipids. The content of PS (22:5_18:2) in porcine milk is higher than in all other samples. Therefore, PS (22:5_18:2) may serve as a biomarker for porcine milk.

### Correlation analysis of the top 20 lipids with the highest differences in positive and negative ion mode

Fig. 3(B) and (C) provide insights into the correlation among metabolic pathways for the identified lipid molecules. Specifically, the top 20 differential lipids in the positive ion mode exhibit a strong positive correlation. Based on previous analyses, the top 20 differential compounds in the positive ion mode exhibit strong species-specificity. Specifically, CHP, MGH, DZD, ALC, and YXP are almost undetectable, yet they are present in higher concentrations in other samples. The associated characteristics of these lipids might suggest that their production is determined by species. Speculatively, based on species features, it is likely related to the fermentation processes of the rumen microbiota. Similarly, most lipids in the negative ion mode demonstrate positive correlations with each other. However, Cer(m40:1), phSM(t36:2), SM(d34:2), SM(d36:2), and PS (22:5_18:2) exhibit positive but relatively weaker correlations with other lipids. Among these five lipids, Cer (m40:1), phSM (t36:2), and SM (d34:2) exhibit a strong correlation with each other, while SM (d36:2) and PS (22:5_18:2) also demonstrate a significant association. Based on previous analyses, the three lipids, Cer (m40:1), phSM (t36:2), and SM (d34:2), share common characteristics in which the concentrations of CHP, HST, GXB, QHY, SNG, NBY, TGB, LCG, and OAS are relatively similar and fall within the median range across all species. Meanwhile, DZD and MGH levels are notably low, and ALC and YXP concentrations are considerably high. For SM (d36:2) and PS (22:5_18:2), they display notably low levels of DZD and MGH, with other sample concentrations exhibiting minimal variations.

As shown in Fig. 3(D), the network interaction map illustrates the interconnectedness of the 20 lipid molecules with the largest differences in both the positive and negative ion patterns. Within the TG molecules, a stronger correlation is observed, which appears to be influenced by the length of the fatty acid chain within the TG molecule. On the other hand, correlations among non-TG lipid molecules primarily revolve around their respective lipid subclasses.

In the positive ion mode, key molecules with high correlation and connectivity to other lipid molecules include TG (6:0_8:0_8:0), DG (22:1e), and TG (4:0_6:0_12:0). These lipids play crucial roles in the network interactions among lipid molecules. In the negative ion mode, Hex1Cer (d19:1_22:0) and Hex2Cer (d19:1_23:0) are key molecules that exhibit high correlation with other lipid molecules, influencing the overall network dynamics.

### Correlation analysis of higher abundant lipid subclasses

To gain insights into the co-regulation of lipids, a chord diagram was employed to visually represent the correlation matrix of lipid-lipid interactions. For this analysis, only lipid pairs with a correlation coefficient |r| > 0.8 and p < 0.05, following the approach described by Aviram et al. (Aviram et al., 2016). The top 10 lipid subclasses, characterized by the highest abundance, were selected for inclusion in the chord diagram. In the correlation chord diagram, the arcs on the outer circle represent lipid subclasses. Colored lines indicate correlations between lipid molecules within each subclass, with the lines color-matched to their corresponding subclass. The thickness of a line originating from one subclass and connecting to another indicates the strength of the correlation; a thicker line suggests a stronger correlation, while a thinner line indicates a weaker correlation.

**Fig. 4:**
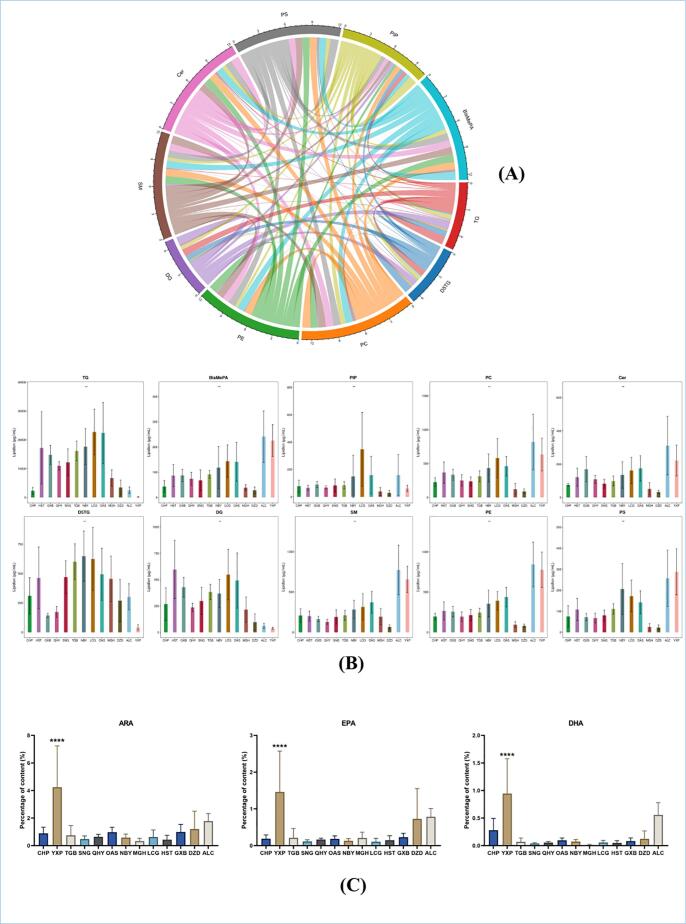
(A) Chord chart of high abundance of lipid subclasses; (B) Bar chart of abundance of lipid subclasses; (C) Bar chart of three EFAs.

As illustrated in Fig. 4(A) and (B), complex correlations were observed within lipid subclasses. Notably, robust associations were identified between TG and DG, D5TG, as well as PIP (phosphatidylinositol (4) phosphate). Similarly, D5TG exhibited strong correlations with DG and PIP. In contrast, PC demonstrated significant correlations with all lipids, except for DG, D5TG, and TG. These findings suggest that DG, D5TG, and TG may exhibit similar abundance trends, while other lipids show strong correlations with these three subclasses. Among the identified subclasses, PIP displayed the highest specificity, correlating with all subclasses examined.

### Analysis of chain length and saturation of TG among different species

**Fig. 5:**
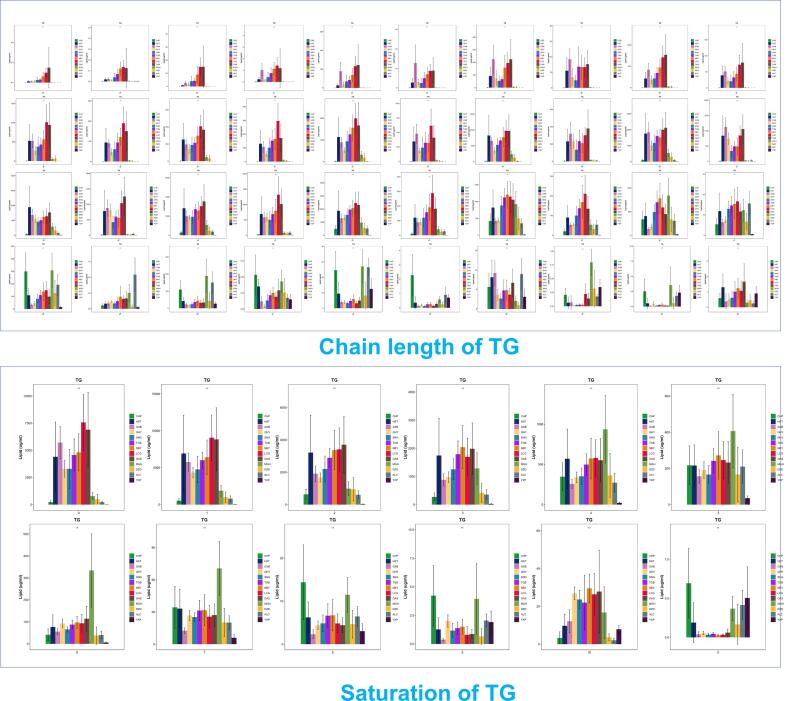
Comparison of TG chain length and saturation levels using a bar chart.

As shown in Fig. 5, The results indicate that in HST, GXB, QHY, SNG, TGB, NBY, LCG, and OAS, the content of TG with carbon chain lengths less than 44 atoms is noticeably elevated. In contrast, the distribution of triglycerides with more than 48 carbon atoms significantly decreases. Among the triglycerides with 48 carbon atoms, CHP and MGH exhibit the highest concentrations. On the other hand, for lipid molecules with a carbon count exceeding 48, the concentration of CHP noticeably increases. Similarly, the levels of ALC, MGH, DZD, and YXP also rise within this range. As the carbon atom count surpasses 52, the content of YXP gradually increases, and when the molecular carbon chain length exceeds 54 atoms, its concentration approaches that of CHP.

From a saturation perspective, HST, GXB, QHY, SNG, TGB, NBY, LCG, and OAS generally have higher levels of low-saturation TG compared to other samples. In CHP, MGH, DZD, ALC, and YXP, the distribution of unsaturated bonds is concentrated in molecules with more than 4 bonds. Specifically, CHP and MGH demonstrate elevated concentrations of TGs with 7–9 unsaturated bonds, implying a similarity between them in this aspect. Most TGs in YXP have more than 8 unsaturated bonds. An important point to note is that TGs with 11 unsaturated bonds are highly expressed exclusively in YXP, MGH, ALC, and CHP. We identified these compounds as TG (18:4_11:4_18:3), TG (16:1_20:5_20:5), TG (16:0_20:5_22:6). Among them, TG (16:1_20:5_20:5) and TG (16:0_20:5_22:6) display higher concentrations in YXP, MGH, ALC, and CHP compared to other species, thus underpinning this finding. Although these lipids are present in trace amounts, they may have significant physiological functions and cannot be substituted by other samples.

### Content analysis of three essential fatty acids (EFAs)

Based on the analysis of the three essential fatty acids (ARA, DHA, and EPA), we observed significant differences in abundance among the species. As shown in Fig. 4(C), YXP exhibited significantly higher levels of ARA, DHA, and EPA compared to CHP. OAS and LCG displayed significantly higher ARA content compared to CHP. In terms of DHA content, CHP had higher levels compared to SNG, QHY, MGH, GXB, and DZD. Overall, considering the concentrations of ARA and EPA, OAS is most similar to CHP. Notably, only YXP exhibited significantly higher DHA content compared to CHP. Regarding EPA content, DZD and ALC had higher levels compared to CHP. This suggests that DZD, ALC, and YXP possess potential as nutritional supplements for infants.

## Discussion

This study comprehensively reveals and compares the lipidomic profiles of milk from different mammalian species. The non-targeted LC-MS/MS lipidomics, combined with base peak chromatography (BPC) and internal standardization, ensures the capture of as many lipids as possible, which is a significant advantage in the current research. Another advantage is the substantial number of samples included in the lipidomic comparisons, which has been rare in previous studies. Our results fill gaps in the milk lipid profiles of certain species and provide a more comprehensive perspective for formula milk design.

### Comparison of lipid content in milk across different species

Significant variations were observed in the distribution of total lipids and lipid subclasses among the different milk samples. As shown in Fig. 1(A), ruminants displayed a higher total lipid content compared to other species, including human milk, which aligns with findings from prior studies (Zhao et al., 2022). This observation can be attributed to the unique diet and digestive system of ruminants. Ruminant animals primarily ingest plant-based feeds characterized by high fiber and low carbohydrate content, often harboring indigenous microbial populations. These microorganisms may influence the transformation of fatty acids. For instance, certain lactic acid bacteria exhibiting probiotic activity have the capability to convert LA into CLA (Aziz et al., 2023). The fermentation of dietary fiber in the rumen results in the production of volatile fatty acids such as acetate, propionate, and butyrate (Bauman & Griinari, 2003). In the gastrointestinal flora of monogastric animals, there exists a lack of the intricate microbial community found in the stomachs of ruminants, which could account for the lower lipid content observed in comparison to ruminants. Camel milk has been reported to contain abundant phospholipids (PLs) and sphingolipids (SLs), constituting over 1 % of total lipids (Bakry et al., 2021). A more recent study indicates that the levels of PLs and SLs in camel milk are close to 10 % (Zhao et al., 2022), while in our research, we discovered higher levels of PLs and SLs in camel milk (Fig. 1(C)). One possible explanation is that the detected lipid species appear more complex due to differences in the mass spectrometry purpose and methodology employed. Due to the crucial role of PLs and SLs in the composition of the milk fat globule membrane, higher levels of these components imply stronger emulsification properties. Smaller fat globules signify a reduced surface area for lipase attachment, indicating a higher concentration of PLs and SLs, thereby facilitating faster digestion (Singh & Gallier, 2017). The size range of camel milk fat globules spans from 1.1 to 2.1 mm, which is lower than those found in buffalo milk (3.9–7.7 mm), cow milk (1.6–4.9 mm), and goat milk (1.1–3.9 mm) (Bakry et al., 2021). This could potentially serve as a plausible explanation for the higher content of PLs and SLs in camel milk compared to human milk. Remarkably, porcine milk exhibited higher levels of PLs and SLs compared to camel milk in this study (Fig. 1(C)). This suggests that porcine milk possesses significant nutritional potential, particularly in terms of phospholipids, making it an excellent candidate as a breast milk substitute.

### Clustering of lipid profiles across different species

The PCA results revealed that DZD and MGH had the closest lipid composition to human milk (Fig. 2(A)). Additionally, trend clustering analysis provided insights into the clustering pattern of lipid molecules. As shown in Fig. 2(B), among the detected lipids, human milk exhibited higher abundance levels in Cluster 1 compared to other species. This cluster was characterized by a dominance of 16-20C fatty acids with high unsaturation, MGH and DZD exhibit the highest content in Cluster4, followed by human milk. Cluster 4 comprises a higher abundance of TG molecules with relatively lower saturation. These clustering is pivotal in comprehending the similarity between DZD and MGH. One plausible explanation could be the absence of rumen hydrogenation in monogastric animals like DZD and MGH, resulting in lower lipid molecule saturation compared to ruminants (Hue-Beauvais et al., 2021). This might explain the proximity of MGH and DZD to human milk. Previous studies have also indicated that MGH and DZD contain levels of PUFAs comparable to human milk (Cimmino et al., 2023, Deng et al., 2022). Both Cluster1 and Cluster4 exhibit an accumulation of PUFAs; however, there are distinctive differences in the specific distribution of lipid molecules between these clusters, possibly associated with the structural variances of these lipid molecules. Further in-depth research is warranted to delve into the origins of lipid molecule distributions. Clusters 2, 3, and 7 showed higher abundance levels in ruminants, which can be attributed to biohydrogenation processes that convert unsaturated fatty acids into saturated fatty acids in ruminant animals (Bauman & Griinari, 2003). Short-chain fatty acids (SCFAs) such as acetic acid, propionic acid, and butyric acid are unique components of ruminant milk fat (Bauman & Griinari, 2003). In ruminants, milk fat synthesis occurs through de novo synthesis in the mammary epithelium and uptake in the circulation, with most SCFAs produced de novo (Lucey et al., 2017). Due to the greater demand for long-chain unsaturated fatty acids to support energy and neural development during human infant development (Einerhand et al., 2023), and considering the maternal intake of nutrients rich in meats (abundant in long-chain fatty acids), human milk typically contains long-chain fatty acids obtained through circulation rather than de novo synthesis (Lucey et al., 2017). Therefore, the mother's need to produce additional short-chain fatty acids (SCFAs) for infant energy maintenance, among other functions, is relatively reduced. In contrast, ruminant animals commonly consume lower-fat plant-based materials, implying a reduced necessity for circulating fewer long-chain fatty acids. To ensure that lipids in milk adequately provide energy for offspring, synthesizing SCFAs from scratch becomes a crucial step. The presence of significant amounts of SCFAs in ruminants’ milk may reflect an evolutionary adaptation. The degradation of SCFAs contributes to milk odor but also exerts significant physiological effects. SCFAs have been shown to reduce allergic reactions in individuals and lower the risk of respiratory tract infections in infants and young children (Kumari & Kozyrskyj, 2017). A notable finding was the presence of numerous long-chain and very long-chain lipid molecules with high unsaturation in porcine milk. These lipid molecules clustered in Cluster 5 and exhibited higher abundance levels. Moreover, porcine milk triglycerides were found to be rich in very-long-chain fatty acids (VLCFAs). VLCFAs are extended by the enzyme elongation of very long-chain fatty acid 4 (ELOVL4) and serve as substrates for the biosynthesis of phospholipids containing VLCFAs (Kawaguchi et al., 2020). The molecular mechanism responsible for the high VLCFAs content in porcine milk remains elusive. However, this elevated content may be attributed to the short gestation period, which necessitates eicosanoids as precursors for synthesizing PGF2α, and the distinctive nutritional demands during the short lactation period. These findings indicate that porcine milk could be a potential source of high-quality fatty acids for infant formula.

### Differential lipid selection and across different species

The top 20 different lipids in the positive ion mode belong to the TG and DG categories (Table 1). Notably, all TG molecules contained at least 2 SCFAs. These lipid molecules were more abundant in ruminants compared to non-ruminants and were scarce in breast milk. This is consistent with the results of trend clustering, implying that these lipid molecules may serve as potential biomarkers for distinguishing ruminant milk from non-ruminant milk. In the negative ion mode, Cer (m40:1) showed significantly higher abundance in YXP and ALC compared to other species (Table 1). Limited studies have been conducted on Cer (m40:1) in lactation stages and pup development in both species. Further investigation is needed to understand the underlying mechanisms behind the high abundance of Cer (m40:1) in camel and porcine milk. The content of PS (22:5_18:2) in porcine milk surpasses that in other species. This lipid molecule comprises 2 PUFAs, and its unique compositional characteristics may suggest beneficial physiological functions. The highest concentration of PS in the human body is in the brain, where it accounts for approximately 15 % of the total phospholipid pool (Buddington et al., 2018). Research has shown that PS esterified PUFA exhibits improved bioavailability compared to TG-associated PUFA (Herrera & Ortega-Senovilla, 2023). This may imply that this molecule plays a crucial role in infant neural development. The correlation heat map and network diagram of these lipids revealed their intercorrelations. Triglycerides (TG) serve as a crucial energy source for infants, and the strong positive correlation observed among these lipid species in TG abundance suggests a shared trend in TG concentration across different mammalian species. Based on our findings, Hex1Cer (d19:1_22:0) and Hex2Cer (d19:1_23:0) showed high connectivity with multiple distinct lipid molecules. Both of these lipid molecules belong to glucocerebrosides. Glucocerebrosides are essential components of glycosphingolipids in cells and play critical roles in various cellular functions, including influencing cytoplasmic proteins, intracellular signal transduction, and maintaining the ordered state of the cell membrane's fluid phase (Reza et al., 2021). Although specific research on the presence of glucocerebrosides in milk fat is lacking, it can be speculated that glucocerebrosides may contribute to the composition of the milk fat globule membrane due to the structural similarities between the cell membrane and the milk fat globule membrane. Further investigation is necessary to explore the secretion mechanism and potential functions of glucocerebrosides in milk.

### Differential profiles of major lipid subclasses and carbon chain length and saturation characteristics in milk across different species

The top 10 lipid subclasses in terms of content can be categorized into two groups: membrane lipids dominated by PLs and SLs, and intramembrane lipids dominated by TGs (Fig. 4(A) and (B)). This is consistent with the research results of Zhao et al. (Zhao et al., 2022). An intriguing finding is the association of phosphatidylinositol phosphate (PIP) with other lipids exhibiting higher abundance, with significantly higher levels observed in a specific species of LCG compared to others. In humans, phosphatidylinositol phosphate kinase (PIPK) catalyzes the phosphorylation of PI to produce PIP, which is involved in immune signaling processes (Beziau et al., 2020). There is a lack of studies on PIP in milk fat, and whether the high content of PIP in LCG serves a specific biological role requires further confirmation. TG plays a crucial role in milk fat as the primary lipid component, contributing to its energy density, texture, and nutritional value. The chain length and saturation of fatty acids in milk fat influence the absorption efficiency and utilization. Fatty acid saturation in milk is closely related to regulatory and structural functions during infant growth, particularly for long-chain polyunsaturated fatty acids (Kim et al., 2017). Previous research indicated the presence of a small amount of medium-chain and a higher proportion of long-chain polyunsaturated fatty acids in human milk (Mazzocchi et al., 2018). Our findings suggest that the predominant chain length of triglycerides (TGs) in human milk falls between 45 and 58 carbon atoms (Fig. 5). Since TG molecules carry three fatty acids, the average chain length for each fatty acid is between 15 and 19, which aligns with prior research. In exclusively breastfed infants, more than 20 % of brain fatty acids are derived from long-chain polyunsaturated fatty acids present in breast milk lipids (Chang et al., 2009). In this study, we observed that horse and human milk exhibited the highest similarity, considering both length and saturation, from an overall perspective (Fig. 5). This implies that at the TG level, MGH and CHP exhibit a high degree of similarity, suggesting that MGH may have the potential to serve as a valuable source of TGs in infant formula.

### A comparative analysis of the three essential fatty acids across different species

Although ARA, EPA, and DHA can be synthesized from their precursors, linoleic acid and alpha-linolenic acid, the conversion rate is low and depends mainly on fatty acid desaturase gene polymorphism, which may not fully meet the physiological needs of infants (Koletzko et al., 2014). Clinical studies have demonstrated that children fed with formulas enriched in DHA, ARA, choline, and sphingomyelin show increased myelin development (Deoni et al., 2018). Previous studies have revealed higher levels of PUFA in sheep's milk (Penhaligan et al., 2022). Our research confirms this finding and identifies three essential fatty acids analyzed, which have higher content than in the milk of other ruminant animals (Fig. 4(C)). However, it is important to note that sheep's milk has lower levels of DHA compared to CHP. Therefore, a formula solely based on sheep's milk may not meet the requirements of infants. Interestingly, our results indicate that porcine milk contains higher levels of some essential fatty acids, compared to human milk. This finding suggests that porcine milk could be a valuable source of essential fatty acids for infant formula. Despite the high content of long-chain and ultra-long-chain fatty acids reported in porcine milk (Mitina et al., 2020), it has been largely overlooked due to traditional consumer perceptions and a lack of milking equipment. In China, pigs are predominantly raised for meat, with limited research on their milk. Our study offers the first comprehensive lipidomic analysis of porcine milk, establishing a foundational understanding for its potential use. Yet, lacking a detailed stereospecific analysis of lipid, we couldn't fully characterize each lipid. Further studies on these lipid structures are warranted, given their potential significance for infant nutrition.

## Conclusion

In this study, we conducted an untargeted lipidomics analysis of milk from 13 animal species and identified 51 lipid subclasses and 2585 lipid molecules. Our analysis provided insights into the lipid metabolism and species-specific lipid profiles of these animals. This study initially explores the total lipid content and compositional characteristics in different mammals. It was found that ruminants have a higher total lipid content compared to pseudo-ruminants and monogastric animals. At the subclass level, pigs and camels exhibit a more unique subclass distribution pattern, specifically with higher phospholipid content. Furthermore, this research investigates the clustering characteristics of species and lipid molecules. In this analysis, donkey and horse milk are considered to be the closest in composition to human milk, revealing that porcine milk contains a significant amount of long-chain polyunsaturated fatty acids. Subsequently, the study further analyzes differential lipid molecules to identify factors contributing to species-specific milk composition. This analysis reveals the characteristic high short-chain fatty acids in ruminant milk, while PS (22:5_18:2) is considered a potential biomarker for porcine milk. Chain length and saturation analysis of TG molecules provide insights into the nutritional significance of lipids, with results indicating that porcine milk has longer chain lengths and higher saturation levels compared to other species, containing higher levels of ARA, DHA, and EPA than breast milk. This study conducted a detailed characterization and comparison of lipid profiles in 13 mammalian species, providing the first comprehensive report of porcine milk's lipid profile. It contributes to enhancing our understanding of mammalian milk lipid information and expands the range of options for exploring more promising formula milk ingredients.

## Financial support

Our scientific research was financially aided by two projects: 1. National Natural Science Foundation of China (No. 32272836); 2. Postdoctoral Science Foundation of China (2021M693859).

## CRediT authorship contribution statement

**Yanzhi Wu:** Methodology, Visualization, Writing – original draft. **Yinggang Sun:** Conceptualization. **Rui Chen:** Formal analysis, Visualization. **Yanjun Qiao:** Software. **Qiu Zhang:** Resources. **Qian Li:** Investigation. **Xiaowei Wang:** Data curation. **Yuan Pan:** Visualization. **Siyi Li:** Data curation. **Zeying Wang:** Funding acquisition, Project administration, Supervision, Writing – review & editing.

## Declaration of competing interest

The authors declare that they have no known competing financial interests or personal relationships that could have appeared to influence the work reported in this paper.

## Data Availability

Data will be made available on request.
