# Supplementary material for: Analysis for lipid nutrient differences in the milk of 13 species from a quantitative non-targeted lipidomics perspective
Source: Food Chem X. 2023 Nov 23;20:101024. doi: 10.1016/j.fochx.2023.101024 (PMC10740049; doi:10.1016/j.fochx.2023.101024)
Supplement: Supplementary data 1 [file mmc1.docx]

**Table 1 Description of samples and their corresponding collection sites**

| **Samples** | **Abbreviations** | **sample collection site** |
| --- | --- | --- |
| Chinese human milk | CHP | Chinese mothers from Shenyang Maternal and Child Care Center |
| Holstein cow milk | HST | Huishan Dairy Group |
| Buffalo milk | GXB | Guangxi Buffalo Research Institute and Nanjing Agricultural University |
| Yak milk | QHY | Qinghai province and Lanzhou institute of husbandry and pharmaceutical science of cass |
| DeZhou Donkey | DZD | East Ajiao Donkey Farm in Fumeng County |
| Mongolian Horse | MGH | Inner Mongolia XilinGol League Science and Technology Association |
| Alxa camel | ALC | Alxa Alpaca Farm in Inner Mongolia |
| Saanen milk goat | SNG | Liaoyang dairy goat farm |
| Nubian milk goat | NBY | Liaoyang dairy goat farm |
| Toggenburg milk goat | TGB | Liaoyang dairy goat farm |
| Liaoning Cashmere goat | LCG | Liaoyang National Core Sheep Breeding Farm |
| Small Tail Han sheep | OAS | Inner Mongolia Horinger County sheep farm |
| YangXiang Pig | YXP | Yangxiang Pig Group |
