# Supplementary material for: Analysis for lipid nutrient differences in the milk of 13 species from a quantitative non-targeted lipidomics perspective
Source: Food Chem X. 2023 Nov 23;20:101024. doi: 10.1016/j.fochx.2023.101024 (PMC10740049; doi:10.1016/j.fochx.2023.101024)
Supplement: Supplementary data 2 [file mmc2.docx]

**Experimental quality control**

This experiment provides a comprehensive evaluation of the stability of the instrument, the reproducibility of the experiment and the reliability of the data quality by means of six quality control elements.

1. Comparison of QC samples Base Peak spectra (BPC)

The BPC plots of the QC samples were compared with each other and are shown in Fig. 1A and 1B. The results show that the peak response intensities and retention times of the QC samples overlapped, indicating good reproducibility of the experiments.
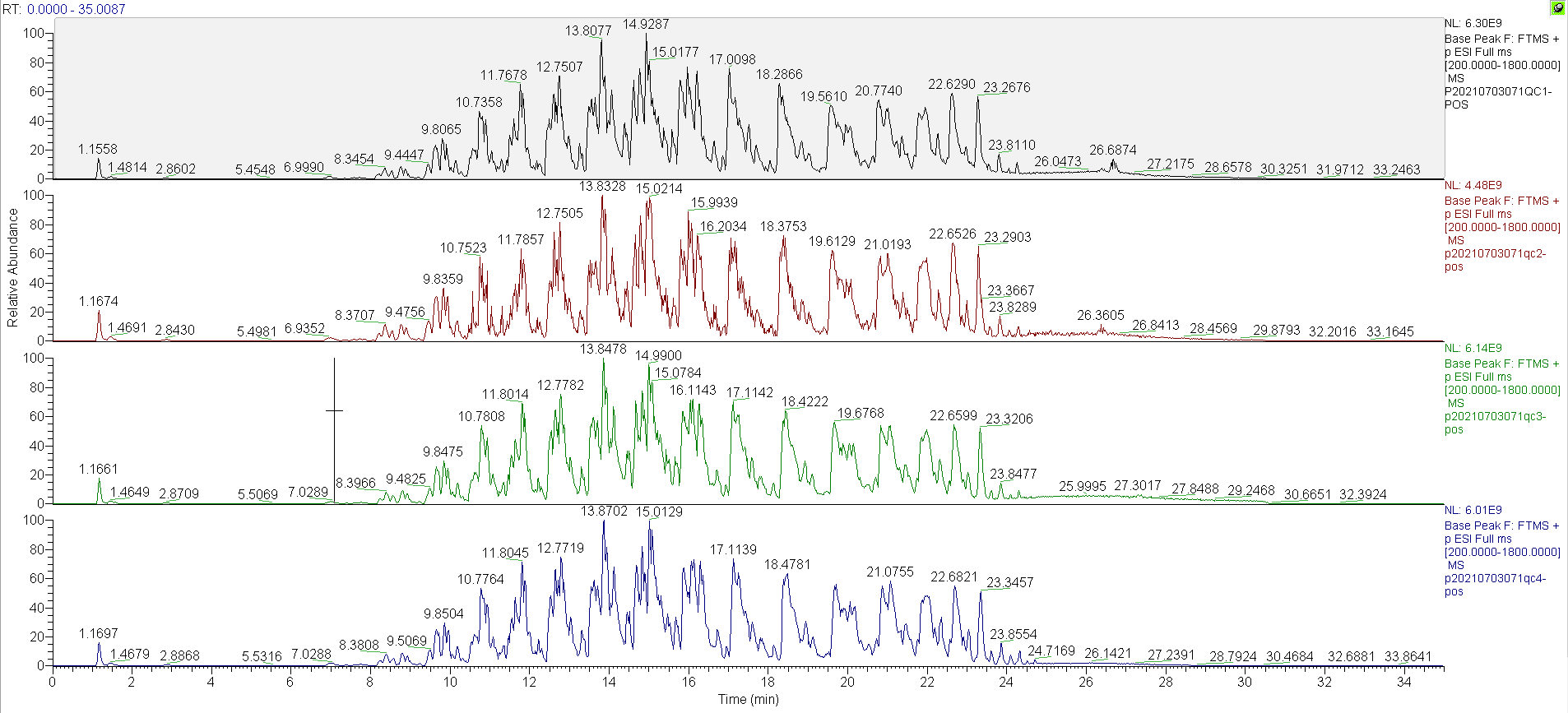


Fig.1A Positive ion mode BPC overlap pattern of QC samples


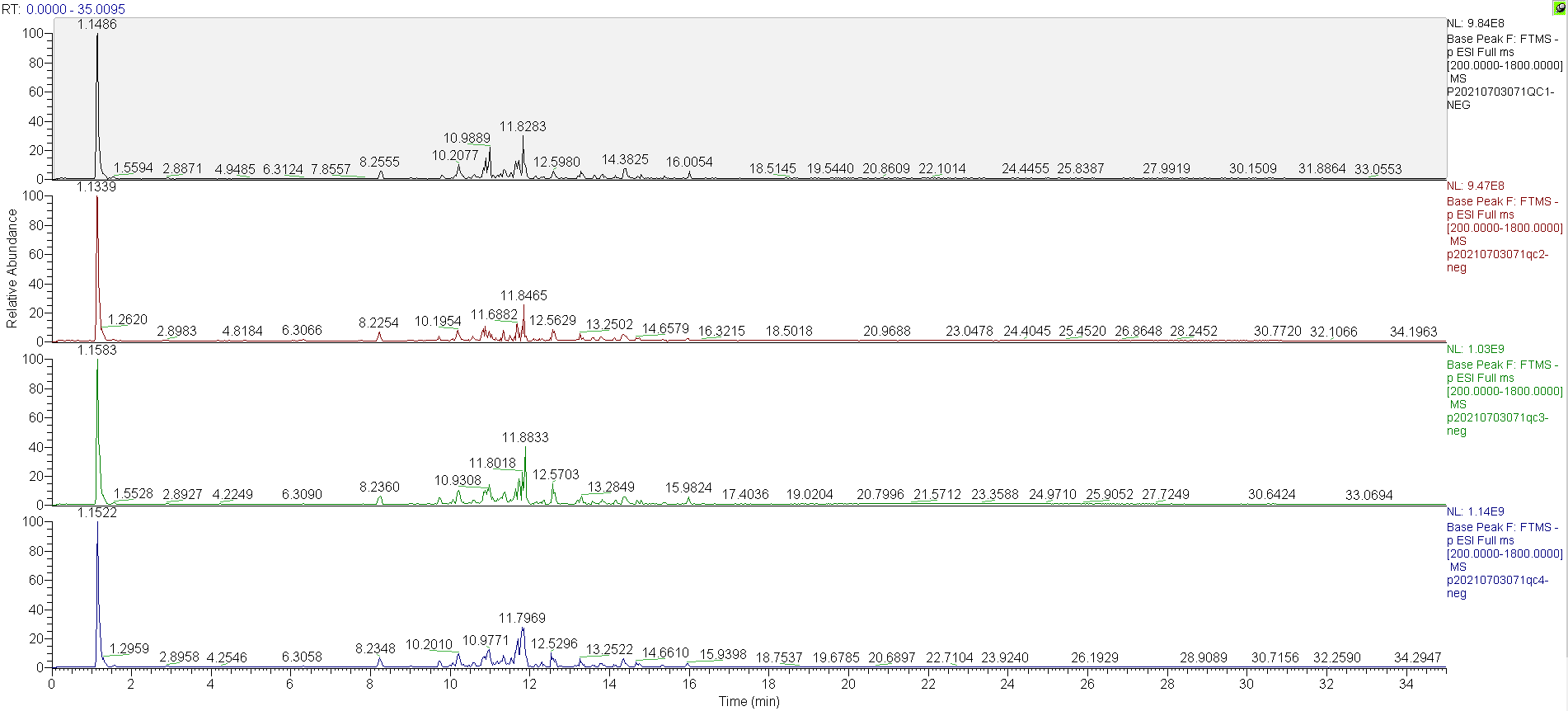


Fig.1B Negative ion mode BPC overlap pattern of QC samples

Note: The horizontal coordinates in the graph indicate the retention time of each peak and the vertical coordinates indicate the intensity values of the peaks.

A Pearson correlation analysis was performed on the QC samples, see Fig. 2. A correlation coefficient greater than 0.9 generally indicates a good correlation. The results of the experiment showed that the correlation coefficients between the QC samples were all above 0.9, indicating good repeatability of the experiment.


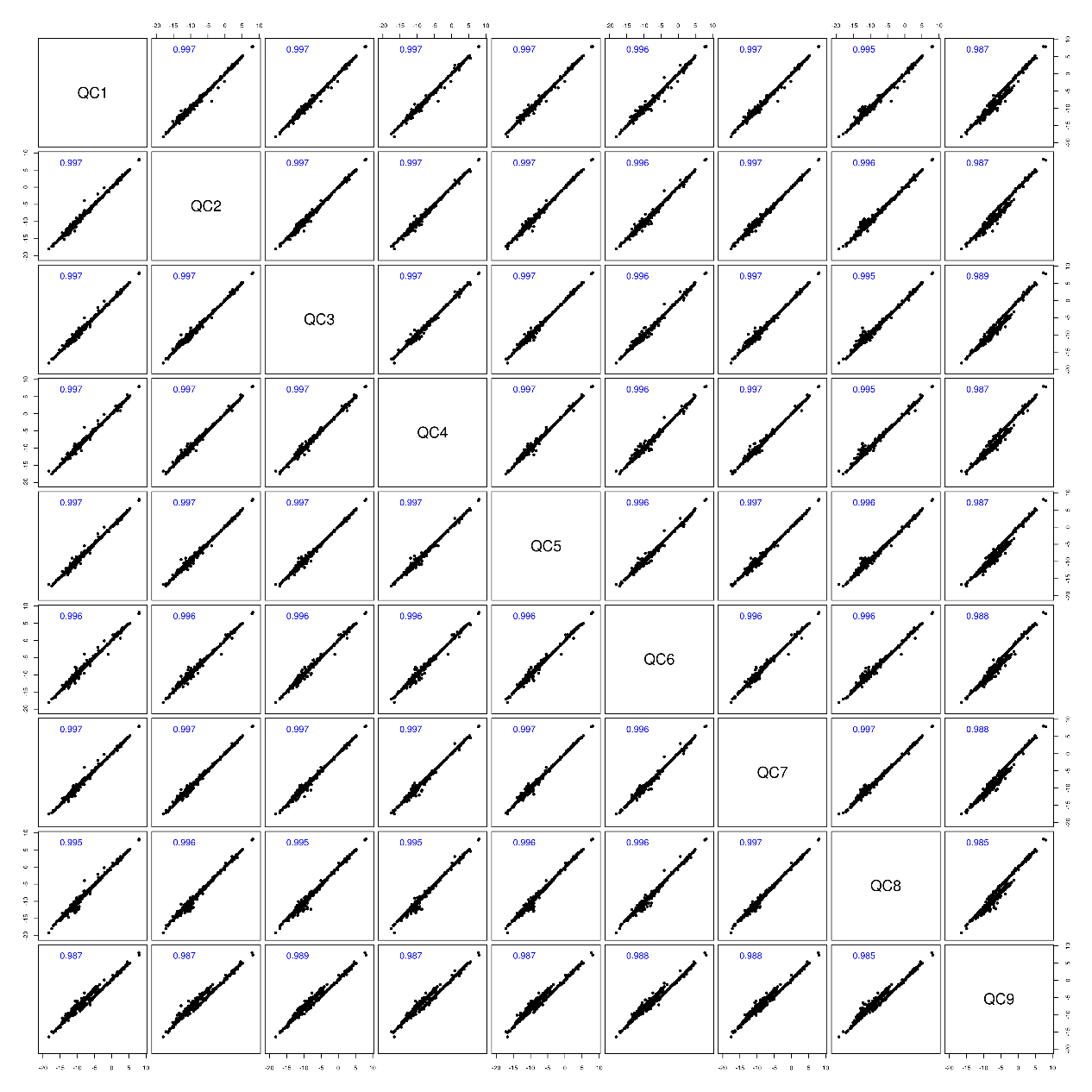


Fig. 2 QC sample correlation map

Note: The horizontal and vertical coordinates in the graph represent the individual QC samples. The points in each cell represent the ion peaks (metabolites) extracted from the QC samples, and the horizontal and vertical coordinates represent the logarithmic values of the signal intensity values of the ion peaks.

1. Overall sample principal component analysis (PCA)

The extracted ion peaks from all experimental and QC samples were subjected to PCA analysis after Pareto-scaling and are shown in Fig. 3. The results show that the QC samples were closely clustered together, indicating good reproducibility of the experiment.


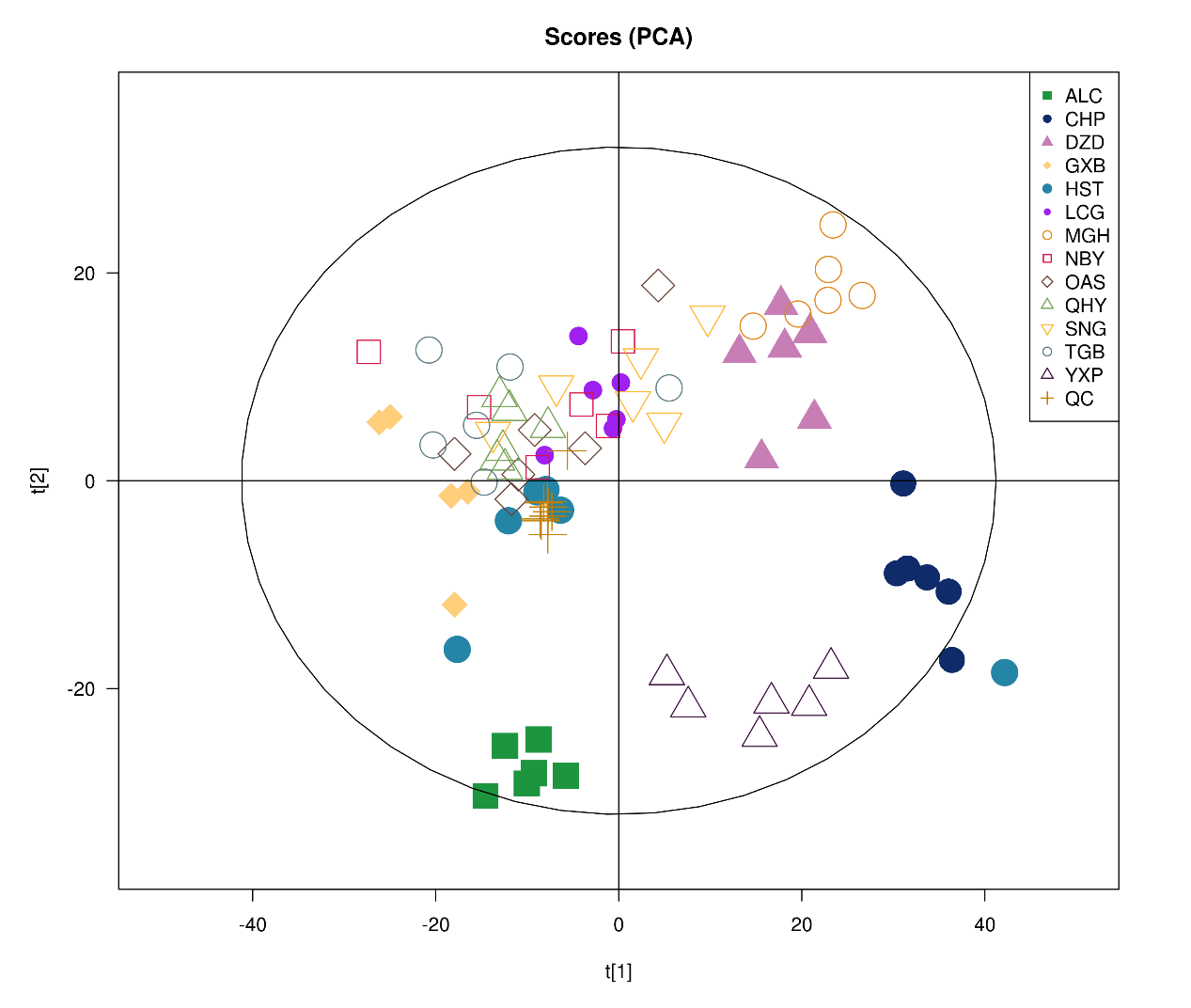


Fig. 3 PCA analysis of the overall sample

Note: In the figure, t[1] represents principal component 1, t[2] represents principal component 2, the degree of aggregation of QC samples reflects how well the experiment is reproduced.

1. Overall sample Hotelling's T2 test

The results of the Hotelling's T2 test are shown in Fig 4. The results of the experiment show that the QC samples were within the 99% confidence interval, indicating that the experiment is Repeatability was good.


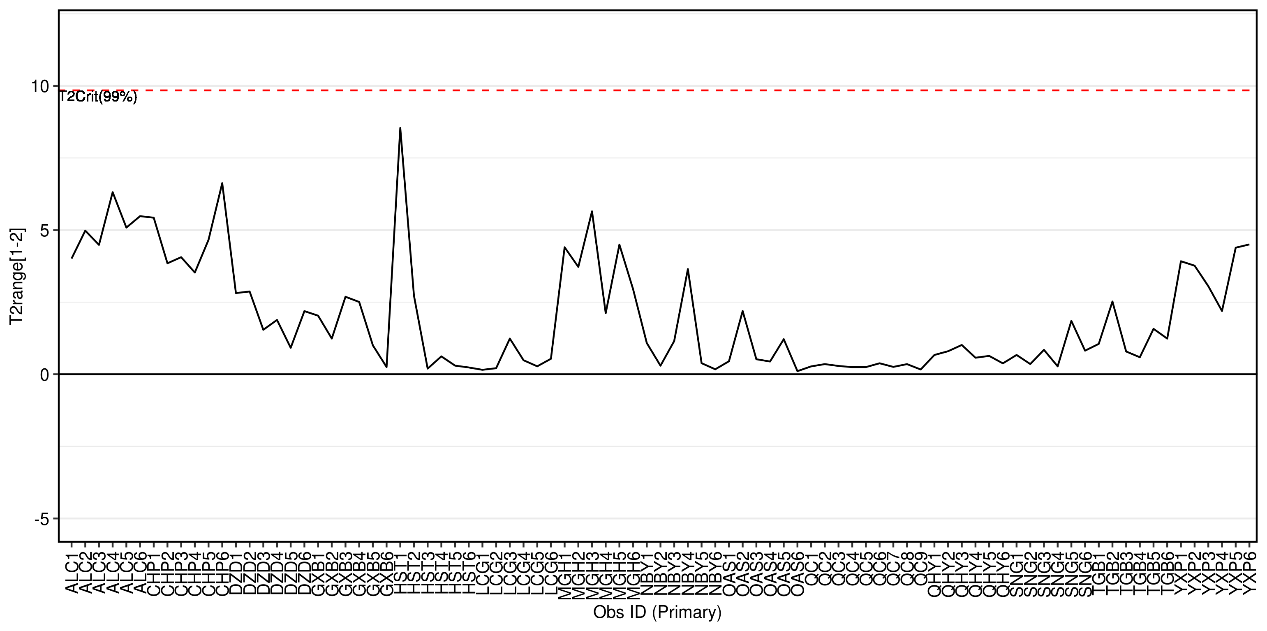


Fig. 4 Hotellings T2 plots for the overall sample

Note: The horizontal coordinates represent all experimental and QC samples, the vertical coordinates reflect confidence intervals, the red line defines the 99% confidence interval range.

1. Multivariate control chart for QC sample

The Multivariate Control Chart (MCC) is a multivariate statistical model based on the ion peaks detected in QC samples and is a quality management tool used to monitor and determine the stability of instrument status.

Each point in the Multivariate Control Chart represents a QC sample and the x-axis is the order in which all QC samples were taken. The points in the chart fluctuate up and down due to fluctuations in instrument status. This is generally within a range of plus or minus 3 standard deviations. The multivariate control chart of the QC samples for this project is shown in Figure 5. The experimental results show that the fluctuations of the QC samples are within plus or minus 3 standard deviations, reflecting that the fluctuations of the instrument are within the normal range and the data can be used for subsequent analysis.
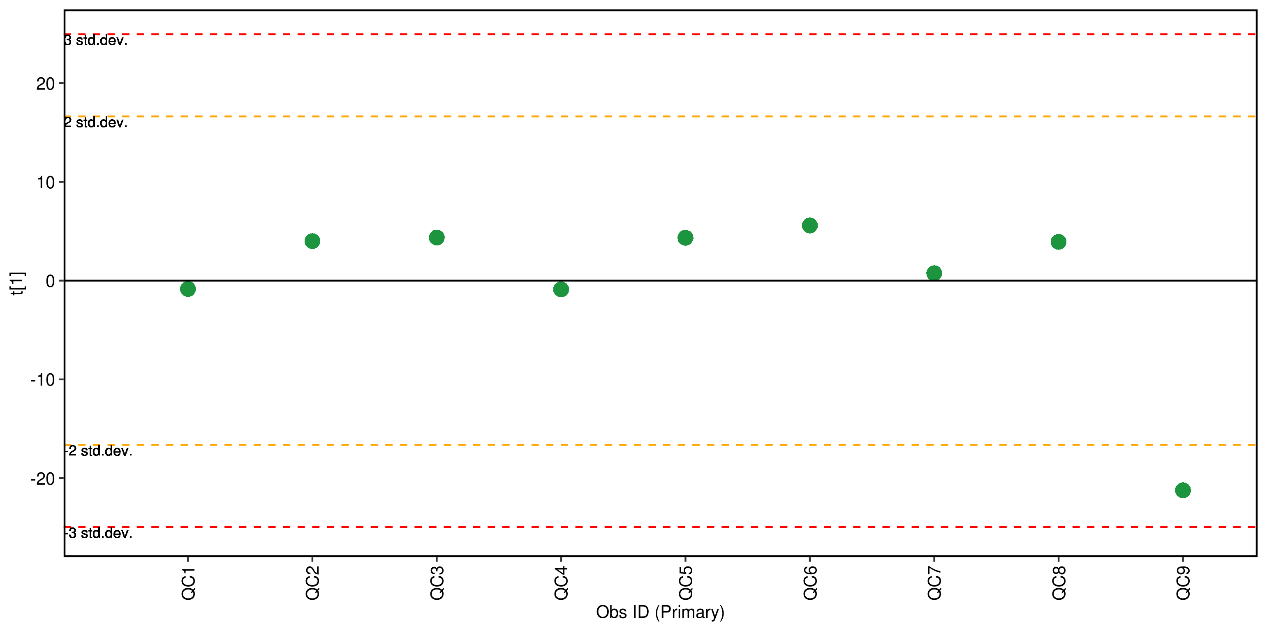


Fig. 5 QC Sample MCC Chart

Note: The horizontal coordinates represent each QC sample, the vertical coordinates reflect the standard deviation, and the yellow and red lines define the range of plus or minus 2 and 3 standard deviations respectively.

1. relative standard deviation (RSD) of QC samples

The smaller the relative standard deviation (RSD) of the ion peak abundance of the QC samples, the better the stability of the instrument, which is an important indicator of the quality of the data. In this experiment, the number of peaks with RSD ≤ 30% in QC samples accounted for more than 80% of the total number of peaks in QC samples, see Fig. 6, indicating that the stability of the instrument analysis system is good and the data can be used for subsequent analysis.


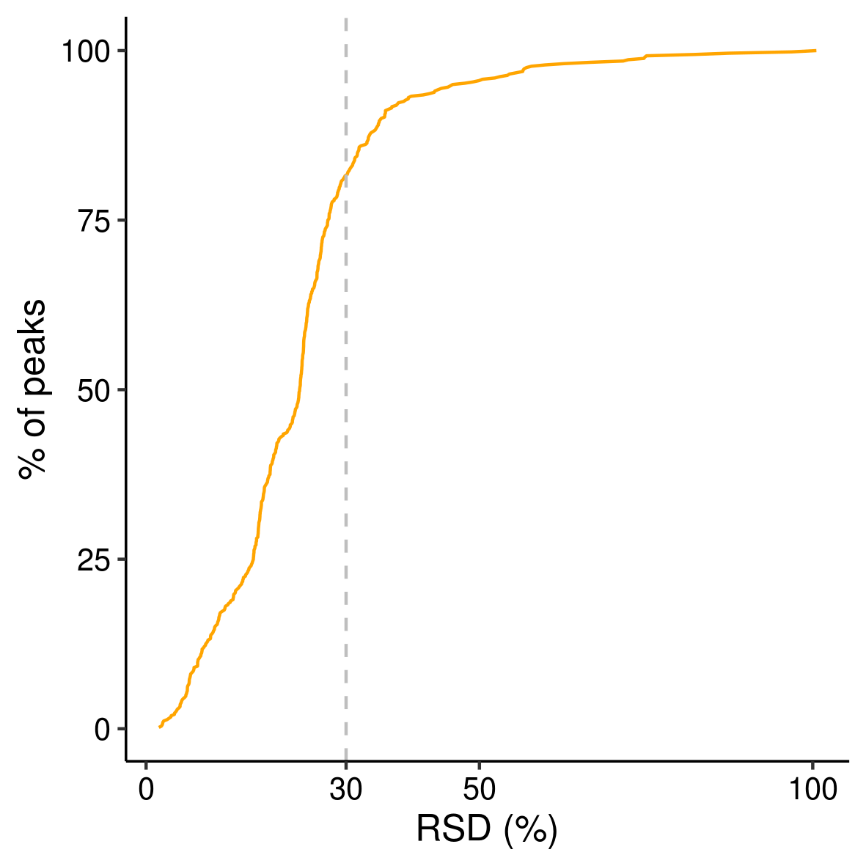


Fig. 6 Relative standard deviation of QC samples
